# Mechanism of anti-dementia effects of mangiferin in a senescence accelerated mouse (SAMP8) model

**DOI:** 10.1042/BSR20190488

**Published:** 2019-09-20

**Authors:** Zhengcai Du, Fangcao Fanshi, Yu-Heng Lai, Jung-Ren Chen, Erwei Hao, Jiagang Deng, Chung-Der Hsiao

**Affiliations:** 1 Guangxi Scientific Experimental Center of Traditional Chinese Medicine, Guangxi University of Chinese Medicine, Nanning 530200, Guangxi, China; 2 Guangxi Key Laboratory of Efficacy Study on Chinese Materia Medica, Guangxi University of Chinese Medicine, Nanning 530200, Guangxi, China; 3 Department of Chemistry, Chinese Culture University, Taipei 11114, Taiwan; 4 Department of Biological Science and Technology College of Medicine, I-Shou University, Kaohsiung 82445, Taiwan; 5 Department of Bioscience Technology, Chung Yuan Christian University, Chung-Li 32023, Taiwan; 6 Center of Nanotechnology, Chung Yuan Christian University, Chung-Li 32023, Taiwan; 7 Center of Biomedical Technology, Chung Yuan Christian University, Chung-Li 32023, Taiwan

**Keywords:** Alzheimer’s disease, amyloid-β, free radical, Mangiferin, SAMP8

## Abstract

Mangiferin (2-β-d-glucopyranosyl-1,3,6,7-tetrahydroxy-9H-xanthen-9-one), a xanthanoid, is one of the major compounds isolated from mango leaves and bark fruit. Previous studies have identified several properties of mangiferin, such as preventing microbial growth, reducing oxidative stress and helping reduce risk of diabetes. The aim of the present study is to explore the potential anti-dementia effects of Mangiferin in a senescence-accelerated mouse prone 8 (SAMP8) mouse model. Morris water maze (MWM) test showed that mangiferin significantly improved the learning and memory retention in SAMP8 mice. In addition, mangiferin reduced the damage in hippocampal neurons and mitochondria, and decreased the expression of amyloid-β (Aβ1-40 and Aβ1-42); however, no influence on the expression of amyloid precursor protein (APP) within the brain of SAMP8 mice. Moreover, Mangiferin inhibited lipid peroxidation (LPO). In conclusion, we provided evidences to show that mangiferin significantly restored the learning and memory impairment in the SAMP8 mouse model, and reduced the pathological injury in hippocampal by modulating lipid oxidation and amyloid-β deposition in the brain.

## Introduction

### The dementia and Alzheimer’s disease problem

Alzheimer’s disease (AD), also known as senile dementia, is an elderly degenerative disease within the central nervous system, which caused progressive memory loss, cognitive dysfunction, judgmental decline, confusion and loss of self-consciousness [1]. AD is one of the most common diseases in modern society that is associated with aging. According to the Alzheimer’s Disease International (ADI), approximately 36 million people worldwide are suffering from AD, and the number of patients doubles every 20 years, which is expected to reach 66 million by 2030, and 115 million people by 2050. Since AD imposes tremendous burden on to healthcare resources, it becomes a worldwide problem that draws concern. Therefore, finding effective prevention and treatment of AD is an imperative and urgent subject [2].

AD was caused by two pathological mechanisms involving τ protein to form paired helical filament (PHF) or neurofibrillary tangles (NFT) in the cerebral cortex area, and then a large number of extracellular β-amyloid (Aβ) aggregation formation of senile plaques (SP) [3]. According to the pathological characteristics, Aβ deposition and the toxic effects of hyperphosphorylated microtubule-associated τ protein (MAPs) became potential targets for AD treatment. Recently, treatment of AD has rapidly developed, including clinical therapies that facilitated brain metabolism, acetylcholinesterase (AChE) inhibitors, metal chelators, antioxidant and anti-inflammatory drugs that were aimed to develop to prevent Aβ deposition. In addition, inhibitors of hyperphosphorylated τ, glutamate receptor modulators, nerve growth factors and estrogen replacement therapies, were also common; however, their clinical efficiencies were still below satisfaction [4].

### The senescence-accelerated mouse prone 8 aging mouse model

The senescence-accelerated mouse prone 8 (SAMP8) is mouse line that displays a phenotype of accelerated aging. It shows degrees of activity loss, hair loss, lordokyphosis and early death. The SAMP8 mouse, was derived from the SAM-P/2 line by Japanese Professor, Toshio Takeda as a rapidly aging dementia mouse model. SAMP8 mice have apparently normal early development with no evidence of growth retardation, yet have a lifespan of only 12 months, with accelerated aging characteristics within 4–6 months of age. The pathological features associated with aging in SAMP8 mouse, showed spontaneous aging within short lifespan with progressive dementia and Aβ deposition, making it a solid animal model for AD research [5]. Research showed that SAMP8 significantly expressed higher level of soluble Aβ in brain, which is related to learning and memory impairment [6]. In addition, SAMP8 mouse in 5-month old showed significantly increased phosphorylated τ, and increase in CDK-5 expression than SAMR1 mouse, which suggested that aging in SAMP8 is associated with τ protein activation [7]. Therefore, these properties make the SAMP8 mice an ideal model for modern study on senile dementia.

### The source and potential usage of Mangiferin

Mangiferin (2-β-d-glucopyranosyl-1,3,6,7-tetrahydroxy-9H-xanthen-9-one), the main bioactive compound extracted from mango leaves, a xanthonoid, a natural flavonoid glycoside, was first isolated from the leaves and bark of *Mangifera indica* [8]. It can also be extracted from mango peels and kernels [9]. Mangiferin showed immunomodulatory, anti-inflammatory [10], anti-lipid peroxidation (LPO) [11], antiviral [12], antitumor [13] and antidiabetic [14] effects. Mangiferin has demonstrated protective function against LPO and damage within brain, by increasing superoxide dismutase (SOD) activity to reduce LPO level. In addition, it also showed protective function against alloxan-associated brain damage. *In vivo* study showed that mangiferin significantly enhanced short-term memory [15]. Moreover, mangiferin promoted secretion of nerve growth factor (NGF) and tumor necrosis factor (TNF)-α (TNF-α) in human neuroblastoma cells, U138-MG, which suggested that mangiferin increased the level of neurotrophic factors and cytokines and may improve recognition memory. Mangiferin was suggested to be a potential neuron protective factor to reduce inflammation and oxidative damages since male Wistar rats fed with mangiferin showed reduction in plasma level of glucocorticoids (GCS), interleukin-1β (IL-1β), TNF-α, cycloxygenase-2 (COX-2), and nitric oxide synthase (iNOS) [11]. In addition, mangiferin significantly enhanced the ability of learning and memory in mice, which suggested its therapeutic effect on Alzheimer dementia [16]. However, Alzheimer’s-related animal model to demonstrate mangiferin function is still required.

### Potential anti-dementia function of mangiferin in SAMP8 model

**Figure 1:**
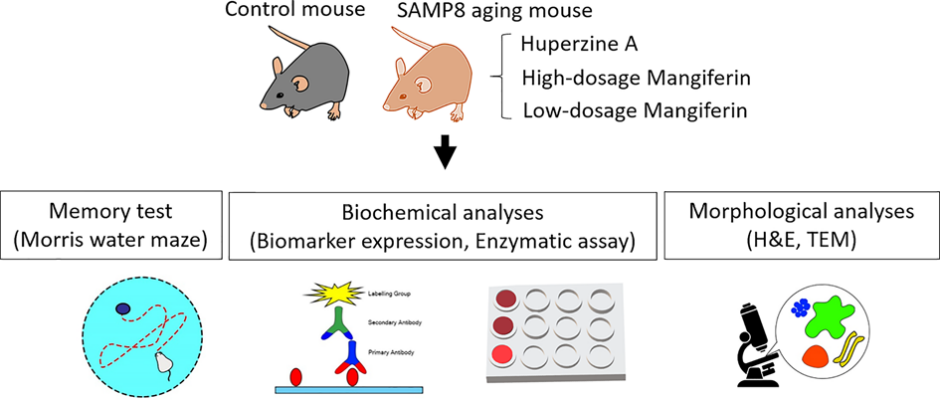
The experimental design of the present study Control and SAMP8 aging mice were used to evaluate the potential anti-dementia effect of mangiferin. After treatment, MWM test was performed to evaluate the learning and memory. By the end of behavioral test, mice were killed and tissues were subjected to biochemical and morphological analyses.

In the present study, we used SAMP8 aging mouse model to study the mechanism of anti-dementia effects of mangiferin (Figure 1). Morris water maze (MWM) test was used to evaluate the impact of mangiferin on memory and behavior in rapidly aging dementia SAMP8 mice [17]. In addition, Crystal Violet staining and immunohistochemistry were used to show pathological changes in hippocampal neurons to detect hippocampal Aβ deposition. Also, brain SOD, malondialdehyde (MDA) and glutathione peroxidase (GSH-Px) activities were detected to fully assess the impact of mangiferin on AD and clarify its mechanism.

## Materials and methods

### Animal grouping and treatment

The accelerated aging and dementia mice model of SAMP8 (aged at 6 months old) was purchased from Department of Laboratory Animal Science, Peking University Medical Department. All the mice were male and were randomly divided into control group, huperzine group A (positive control group), mangiferin high-dose group, and mangiferin low-dose group. Mangiferin extracted compound was a gift from Dr. Xuejian Li from Guangxi University of Chinese Medicine. Mangiferin was dissolved in double-distilled water and mixed by gentle shaking. Huperzine A was purchased from Taloph Pharmaceutical (lot number H11940151, Henan, China). Mice were subjected with 100 or 200 mg/kg/day as low and high doses mangiferin, respectively. Huperzine A was subjected in mouse with 0.05 mg/kg/day. The control group was fed with distilled water with the same volume in treated groups. Mice were orally dosed once a day for 60 days with volume of 0.02 ml/g and double-distilled water was provided along with oral administration.

### Specimen collection

After 60-day treatment, behavioral examination was conducted, then killed and decapitated to remove the intact brain tissue. Tissue was separated into two symmetrical parts, one for immunohistochemistry and one for biochemical analysis. Tissue was fixed with 4% paraformaldehyde in PBS before staining. Another half part of the tissue was for detection of enzyme in nerve and brain tissue after weighing. Tissue was directly transferred to 2-ml glass homogenizer tube with PBS (4 ml/g tissue), fully homogenized for 6–8 min, and centrifuged. Tissue was stored at −80°C before analysis.

### MWM

We used MWM to determine the memorizing status between aging and normal mice. After continuous drug administration for 60 days, we transferred mice to animal behavioral core facility to test adaptation/swimming ability test by day 61, spatial navigation test by days 62–66 and spatial exploration test by day 67. The size for MWM is 200 cm diameter, 50 cm height and 30 cm depth. The circular pool was filled with distilled water and kept at 23–25°C. The pool was divided into four quadrants with equal area. A transparent plastic platform (4.5 cm in diameter and 14.5 cm in height) was centered in the second quadrant of the pool. For adaptation/swimming ability test, the transparent plastic platform was removed and let the tested mouse can freely explore the environment. The locomotion activity was recorded for 2 min and the xy trajectory was tracked by using WMT-200 software (Chengdu Techman Software Co. Ltd, Chengdu, China). For spatial navigation test, a transparent plastic platform was put in the center point of the second quadrant and kept its position 1 cm under the water level. The mouse was transferred into water maze at the random entry point and their locomotion was videotaped for 60 s. The time for mouse to find the stage was in this 60 s. Test is defined as the escape latency. For each daily trial, the mouse was placed into the water maze at one of three randomly determined locations and was released to find the hidden platform. After the mouse found and climbed on to the platform, the trial was stopped. The time for maximum trial length was 60 s. If animals did not locate the platform within 60 s, they were guided to the platform, and kept on the escape platform for 10 s and noted as 60 s escape latency. For spatial exploration test, the transparent plastic platform was removed, and the mouse experienced the previous test was transferred to water maze and their locomotion activity was videotaped for 120 s. The time percentage which mouse swimming in the original quadrant and the times mouse crossed the stage was recorded.

### H&E staining and immunohistochemical staining

For paraffin section, brain tissues were fixed in 4% paraformaldehyde/PBS for 1 day and then dehydrated in ascending ethanol, cleared with Neo-clear (Merck) and embedded in Paraplast Plus/Paraplast HM (Leica) in a 7:3 ratio (vol/vol). The paraffin embedded tissues were sectioned at 5-μm intervals with a rotational microtome (HM360, Microm) and then stained with H&E staining kit according to the manufacturer’s instructions (Merck). For immunohistochemistry, the deparaffined slides were treated for antigen retrieval by incubating with 1 mM EDTA (pH 8.0) solution at 95°C for 30 min. The slides were then subjected to antibody staining with primary antibodies as follows: anti-amyloid precursor protein (APP, 1:200, BA0580, Boster Biological Technology, Wuhan, China), anti-Aβ1-40 (1:200, AP481P, AnaSpec), anti-Aβ1-42 (AP482P, AnaSpec), goat anti-rabbit IgG (1:500, AP162P, AnaSpec). The HRP–conjugated goat anti-mouse IgG secondary antibodies were applied at 1:500 dilution and the immunoreactive signals were developed using DAB as substrate (SK-4100, Vector Laboratories). Finally, the slides were counterstained with Hematoxylin for 5 s and mounted using Neo-Mount (Merck). Olympus GX71 wide field high pixel with displayed emblem mirror was used to observe and capture images of mouse hippocampal region. Images were processed with Image-Pro Plus 7.0 image software.

### Transmission electron microscopy

Brain tissues were dissected and cut into small pieces (2 mm × 2 mm) and fixed in paraformaldehyde. The tissues were then embedded in Epon and dissected into 70-nm thick sections. Tissue sections were put on the grids (three to four sections on one grid) and stained with uranyl acetate and lead citrate for 3 min, sequentially. Grids were washed with water for 1 min and dried completely. Mitochondrial morphology in the hippocampus CA1 region was captured by using digital camera on transmission electron microscopy (TEM) with identical magnification.

### Measurement of biomarker

Brains were removed and homogenized at medium speed with Bullet blender tissue homogenizer (Next Advance, Inc., NY, U.S.A.) with 50 volumes of (v/w) ice-cold PBS. Samples were further centrifuged at 13000×***g*** for 10 min and the crude extract was stored at −80°C until further use. The relative activities of total SOD (t-SOD) (A015-1), GSH-Px (A005) and MDA (A003-1) in mouse brain were measured by using enzymatic kits (Nanjing JianCheng Bioengineering Institute, Nanjing, China) according to the manufacturer’s instructions.

### Statistical analysis

Data for each group were expressed as mean ± SEM and were compared by using one-way ANOVA with Tukey’s post-hoc analysis. All statistics were plotted and compiled by using GraphPad Prism (GraphPad Software version 7 Inc., La Jolla, CA, U.S.A.). Significant difference between control and treated groups was set at *P*-value <0.05.

## Results

### Effect of mangiferin on learning and memory in SAMP8 mice

**Figure 2:**
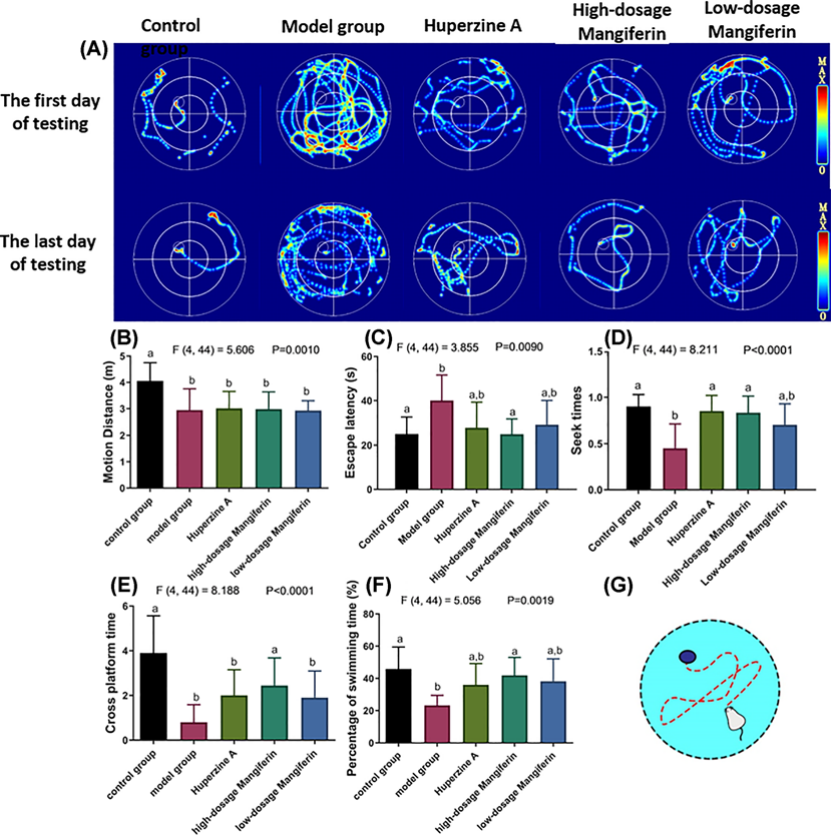
Learning and memory for SAMP8 aging mouse with or without mangiferin treatment by MWM (**A**) The locomotion trajectory of the control and the SAMP8 aging mouse in MWM. The quantitative comparison of (**B**) Motion distance, (**C**) Escape latency, (**D**) Seek times (the time for mouse to find the platform successfully), (**E**) Cross platform times (the incidence for mouse swimming cross the platform), (**F**) Percentage of swimming time for the SAMP8 aging mouse with or without mangiferin treatment. (**G**) The basic principle and setup of MWM (*n*=9–10, mean ± SEM). Different labels above columns in (**B–F**) indicate a significant difference tested by one-way ANOVA and post hoc test between different experimental groups with *P*<0.05. The F and *P-*values for one-way ANOVA test were also highlighted in the top panel in (B–F). Letters ‘a’ and ‘b’ represent different statistical differences within *P*<0.01. ‘a,b’ indicates no significant difference between both ‘a’ and ‘b’ bars.

Water maze was designed to exam spatial learning for rodent and evaluate the hippocampus-dependent navigation and memory (Figure 2G) [17]. The normal control, the SAMP8 (model group), the SAMP8-huperzine A (positive group) and the SAMP8-mangiferin groups were subjected to MWM test. Compared with the model group, the swimming distance was significantly decreased in the SAMP8 mice after feeding with either huperzine A or mangiferin (Figure 2A,B). Generally, the time interval spending on swimming toward the maze platform is referred as the learning and memory abilities (Figure 2C–F). We observed that mice with high dose of mangiferin treatment restored the ability to navigate the platform, indicating that mangiferin is beneficial to the learning and memorial memory abilities.

### The effect of mangiferin on hippocampal pathology

**Figure 3:**
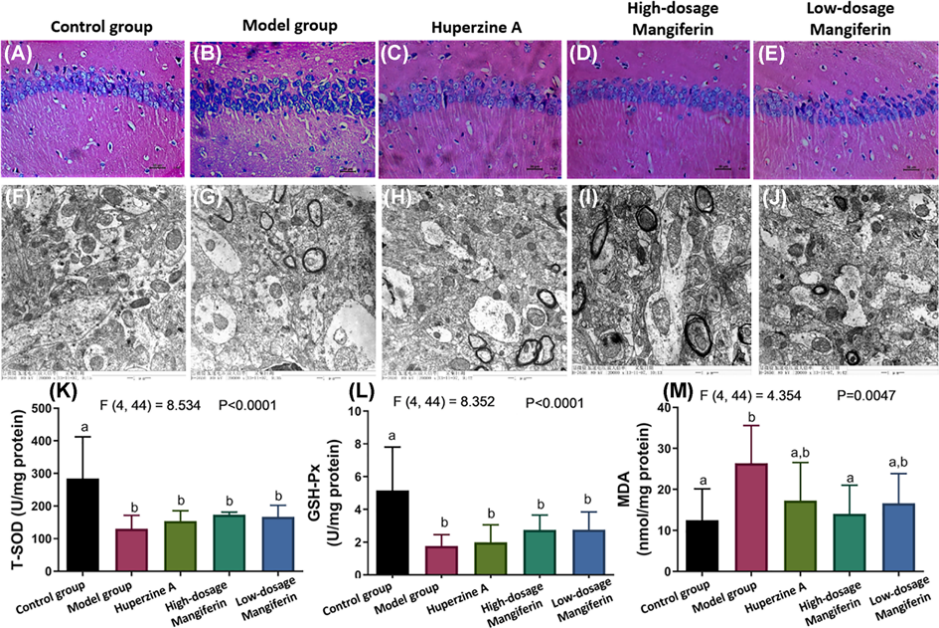
The histology of SAMP8 mouse with or without mangiferin treatment (**A**–**E**) H&E staining showing the tissue morphology within hippocampus CA1 area. (**F**–**J**) Transmission electron microscopy showing the tissue morphology within hippocampus CA1 area. (**K**) Detection of t-SOD activity in the SAMP8 aging mice with or without mangiferin treatment. (**L**) Detection of GSH-Px activity in the SAMP8 aging mice with or without mangiferin treatment. (**M**) Detection of LPO marker of MDA in the SAMP8 aging mice with or without mangiferin treatment (*n*=9–10, mean ± SEM). Different labels above columns in (B–F) indicate a significant difference tested by one-way ANOVA and post hoc test between different experimental groups with *P*<0.05. The F and *P*-values for one-way ANOVA test were also highlighted in the top panel in (K–M). Letters ‘a’ and ‘b’ represent different statistical differences within *P*<0.01. ‘a,b’ indicates no significant difference between both ‘a’ and ‘b’ bars.

To detect the neuronal alignment within hippocampus area, it suggested us pathological status of neurons and evaluated the brain function. Generally, neurons within the hippocampus were tightly aligned with similar size neurons, without tangled and vacuoles (Figure 3A). However, neuron were thinning, interrupted, shrinkage, nuclear pyknosis and deeply stained with H&E staining in the SAMP8 model group (Figure 3B). Compared with the model group, the SAMP8 treated with either huperzine A or mangiferin, the cerebral cortex and hippocampus morphologies showed various degrees of recovery. Neurons were arranged orderly into a compact structure, uniformly distributed and showed normal histological staining (Figure 3C–E). Furthermore, electron microscope observation showed that mitochondria within brain were oval and intact with a clear structure, arranged orderly ridge, no swelling and no sign of vacuolar degeneration in the control group (Figure 3F). On the contrary, swelling, thickening in the membrane, fuzzy, crest and fractured morphology were observed in the mitochondria of the brain in the SAMP8 model group (Figure 3G). Nonetheless, when SAMP8 mice were treated with huperzine A or mangiferin, the structure of the mitochondrial crista was restored to a normal, orderly and mild swelling morphology (Figure 3H–J). It suggests that mangiferin have a potential to restore the damages in hippocampal neurons and mitochondria.

### The effects of mangiferin on free radical metabolism in the SAMP8 mice brain

The oxidative stress was considered associated with aging, which t-SOD and GSH-Px expressed lower along lifespan [18]. In the control group, the content of t-SOD and GSH-Px expressed was significantly higher than the SAMP8 aging group (Figure 3K,L). After treating with either huperzines A or magiferin, the t-SOD and GSH-Px were at similar level to control mice (Figure 3K,L). MDA is a marker to evaluate LPO level in tissues [19]. We observed that the MDA content was significantly reduced after mangiferin treatment, suggesting mangiferin showed effect on reducing the lipid peroxidative stress (Figure 3M).

### The effect of mangiferin on expressions of APP and Aβ in the SAMP8 mice brain

**Figure 4:**
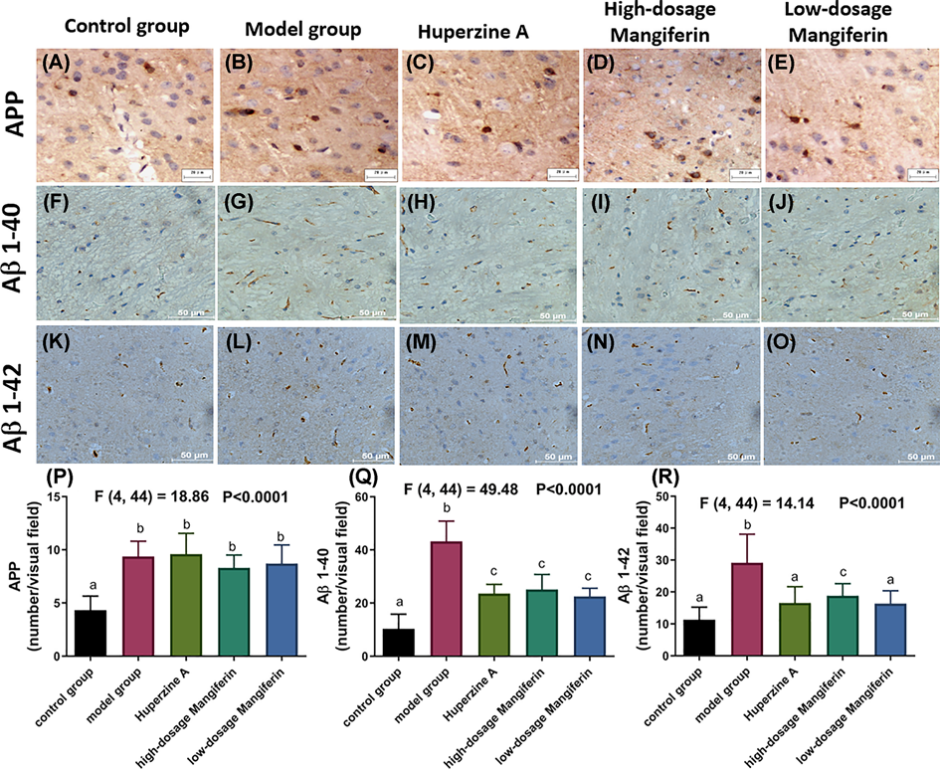
The immunohistochemistry analysis of APP and Aβ proteins for SAMP8 aging mouse with or without mangiferin treatment (**A**–**E**) Immunostaining for APP protein expression in control (A), model group (B), huperzine A-treated group (C), high-dose mangiferin-treated group (D) and low-dose mangiferin-treated group (E). (**G**–**J**) Immunostaining for Aβ1-40 protein expression in control (**F**), model group (G), huperzine A-treated group (H), high-dose mangiferin-treated group (I) and low-dose mangiferin-treated group (J). (**K**–**O**) Immunostaining for Aβ1-42 protein expression in control (K), model group (L), huperzine A-treated group (M), high-dose mangiferin-treated group (N) and low-dose mangiferin-treated group (O). Quantitative comparison of APP (**P**), Aβ1-40 (**Q**) and Aβ1-42 (**R**) protein expression of the SAMP8 aging mice with or without mangiferin treatment (*n*=9–10, mean ± SEM). Different labels above columns in (P–R) indicate a significant difference tested by one-way ANOVA and post hoc test between different experimental groups with *P*<0.05. The F and *P*-values for one-way ANOVA test were also highlighted in the top panel in (P–R). Letters ‘a’, ‘b’ and ‘c’ represent different statistical differences within *P*<0.01.

The Aβ precursor, APP, Aβ1-40 and Aβ1-42 were mainly distributed in the parietal cortex, hippocampus CA1 and CA3 areas [20]. Compared with control group (Figure 4A), we observed that the expression of APP significantly accumulated within hippocampus of the SAMP8 mice (Model group, Figure 4B). Compared with control group (Figure 4F,K), we observed that the expression of Aβ1-40 and Aβ1-42 proteins were significantly elevated in the SAMP8 model group (Figure 4G,L). When the SAMP8 mice were treated with either huperzine A or mangiferin, the amount Aβ1-40 (Figure 4H–J,Q) and Aβ1-42 (Figure 4M–O,R) were reduced, suggesting that the mangiferin is effective in ameliorating Aβ formation. On the contrary, APP level was not significantly changed when the SAMP8 mice were treated with either huperzine A or mangiferin (Figure 4C–E,P).

## Discussion

Dementia is a chronic progression of mentally functional declines that causes behavioral disorder. Among the dementia cause by aging, AD is one of the most common form in modern society [21]. The MWM has been well-developed for evaluating the relationship between performance and neurological function or myriad drug effects [22]. Moreover, SAMP8 mice model has been well established to study aging-related neurological disorder in memory and learning [23]. Generally, several symptoms were considered as diagnosis markers of AD-associated incidence in SAMP8 mice, such as Aβ-like granular structure, sponge degeneration, accumulation of lipopigments and cortical atrophy. Among the morphologies, phosphorylated τ protein in patients of AD has not been demonstrated in SAMP8 mice model [23]. In our study, the aging-related degeneration hallmarks, neurofiber tangles and senile plague within hippocampus associated with memory and learning were observed. In our study, we found that SAMP8 mice treated with mangiferin demonstrated better swimming navigation and learning ability in MWM test. Along with the pathological recoveries after mangiferin treatment, it suggested to us the mangiferin may have potential to restore the neuronal damages and death of neuronal cells, which may help human free from elderly dementia.

Since Aβ deposition is the leading event in AD developing, combined with the recognition impairment, it became the typical diagnostic marker in AD. With the degree of aggregation and deposition of Aβ, it helps to diagnose AD with greater accuracy [24]. Neurotoxic Aβ, a polypeptide containing 37–49 amino acid residues, the primary component of amyloid plaques found in the brains of AD patients, was sequentially proteolyzed from APP. APP is an integral membrane protein that is expressed in many tissues and concentrated in the synapses of neurons. However, the precise role of APP is still not clear with only implication as a regulator of synapse formation [25], neural plasticity [26] and iron export [27], and neuronal cell growth [28]. Furthermore, the intercellular C-terminal domain of APP was served as transcriptional factor and associated with cell sorting [29]. While APP was existed in neuron in a great amount and metabolized quickly, two of proteolytic isoforms, Aβ-40 and Aβ-42, were believed to be the most typical sign of AD [30]. Therefore, in this study, we observed and quantified the expression level of Aβ-40 and Aβ-42 to investigate the effect of mangiferin on dementia-related Aβ accumulation.

Increasing evidence indicated that free radical-induced oxidative damage is one of stresses that result in AD. Unbalanced oxidants reacted with oxidizable lipid within the brain renders the tissue vulnerable [31]. Therefore, we decided to measure the level of t-SOD and GSH-Px to test the hypothesis that mangiferin extract can restore AD-like symptom through lowering the aging-induced oxidative stress. Although our results showed that mangiferin treatment is unable to elevate t-SOD and GSH-Px levels in SAMP8 aging mouse model; however, it reduced the MDA content in the brain, which is suggesting the LPO level has been successfully attenuated.

**Figure 5:**
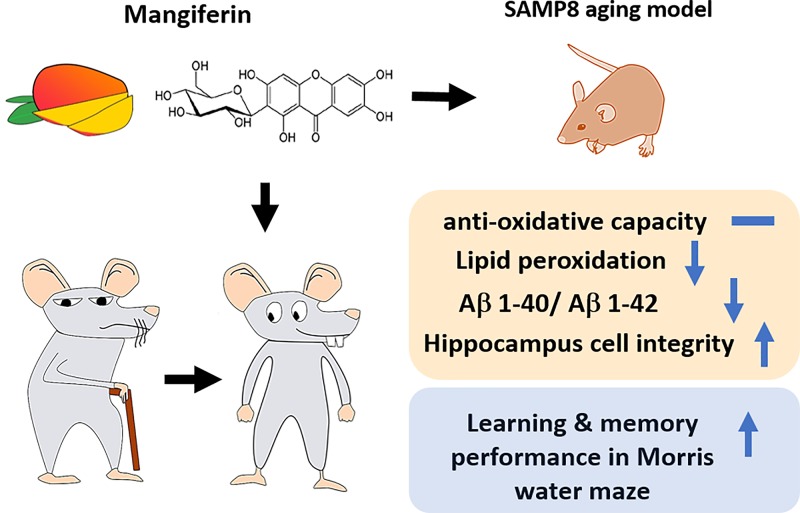
Schematic representation of the obtained results and the protective effects of mangiferin to reduce memory loss in SAMP8 mouse model The morphological and biochemical changes are summarized and highlighted in orange color-shaded box. The behavioral changes are summarized and highlighted in blue color-shaded box.

To sum up, we applied SAMP8 aging mice model to evaluate the potential function of mangiferin’s effects on preventing neurological damage and dementia. We demonstrated that bioactive component in mangiferin may improve learning and memory during MWM test, keep hippocampus cell integrity, reduce brain Aβ species accumulation and reduce brain LPO level, which indicated great potential to be a therapeutic alternative to restore AD-induced neurological defects (summarized in Figure 5).

## Abbreviations

AD: Alzheimer’s disease
APP: amyloid precursor protein
Aβ: β-amyloid
GSH-Px: glutathione peroxidase
LPO: lipid peroxidation
Mangiferin: 2-β-d-glucopyranosyl-1,3,6,7-tetrahydroxy-9H-xanthen-9-one
MDA: malondialdehyde
MWM: Morris water maze
SAMP8: senescence-accelerated mouse prone 8
SOD: superoxide dismutase
TNF-α: tumor necrosis factor
t-SOD: total SOD
